# Assessing breast self-examination knowledge, attitude and practice as a secondary prevention of breast cancer among female undergraduates at the University of Dodoma: a protocol of analytical cross-sectional study

**DOI:** 10.3389/fepid.2024.1227856

**Published:** 2024-05-30

**Authors:** Glory Masawa, Joanes Faustine Mboineki

**Affiliations:** Department of Nursing Management and Education, School of Nursing and Public Health, The University of Dodoma, Dodoma, Tanzania

**Keywords:** breast self-examination, knowledge, attitude, breast cancer, secondary prevention

## Abstract

**Background:**

Breast cancer is a global disease affecting an enormous number of women worldwide and a primary cause of cancer-related deaths in women. In Tanzania, women's breast cancer is the second-ranked among all types of cancers, with an incidence of 15.9%. Despite the Breast Self Examination (BSE) being one of the secondary prevention of breast cancer and an important measure for early diagnosis of breast cancer, little is known whether women could practice routine BSE.

**Objectives:**

The study aims to Assess breast self-examination (BSE) knowledge, attitude, and practice among female undergraduates at the University of Dodoma.

**Methodology:**

An institutionally-based analytical cross-sectional study will be carried out in the Dodoma region of Tanzania through a validated questionnaire. The study will involve 384 female undergraduate students aged 18 years. The data will be entered and analyzed in the Statistical Package for Social Sciences (SPSS) Version 25. Descriptive analysis (frequencies and percentages) and inferential statistics [Cross-tabulation, Chi-square (*χ*^2^), and Regression analysis] will be used.

**Conclusion:**

The practice of BSE among women remains unsatisfactory, which is highly linked to the lack of BSE knowledge. The deficit knowledge is on how to perform BSE, the benefit of BSE, and the interval of doing BSE. Most of the women seem to possess a positive attitude towards BSE.

## Introduction

Breast cancer is a global disease affecting an enormous number of women worldwide and a primary cause of cancer-related deaths in women (1). According to World Health Organization (WHO), 2.3 million women worldwide were diagnosed with breast cancer in 2020, among them 685,000 died (2). In developing countries, the prevalence of breast cancer is increasing due to unhealthy lifestyles like smoking, physical inactivity, calorie-dense food consumption, changes in childbearing and breastfeeding, and exogenous hormonal intake (3).

Breast cancer mortality is secondary to late diagnosis, causing poor outcomes of management and eventually death (4). Regular BSE is among the options to prevent breast cancer deaths (5). BSE is the secondary prevention of breast cancer (6). BSE is a cheap and affordable secondary prevention of breast cancer among third-world countries that aids in the detection of tumors and abnormalities predisposing to malignant tumors compared to Mammography (4).

In Ethiopia, BSE prevalence is as low as 15.2% (7). In India, 57.5% of nursing students were aware of BSE, only 5.3% performed BSE and among them, 6.6% knew the correct BSE testing procedure (8). It is reported that 44% of girls had inadequate knowledge of BSE and 54% had unfavourable attitudes (9). In another study it is indicated that 66.3% of participants had poor knowledge about breast self-examination, however, 95.3% had favourable attitudes.

BSE which aims to detect breast abnormalities involves five steps; ① examining breasts in a mirror while placing hands on hips and maintaining a straight back ② raising arms to check for the same changes in the breasts ③ looking for breast fluid warning signs while looking in the mirror ④ examine for breast lumps while lying down using the right hand to feel left breast while the left hand behind the head and vice versa and ⑤ examining breasts while sitting or standing using firm smooth touch with fingers like step three (10). The reported factors influencing BSE involve the family history of breast cancer, knowledge of BSE, self-efficacy in performing BSE, and information sharing from a health professional and media (11).

In Tanzania, women's breast cancer is the second-ranked among all types of cancers, with an incidence of 15.9%, and newly diagnosed cases of 3,992 women out of 29,883,105 population (12). More than 80% of breast cancer patients receive their diagnosis at stage III or IV (13). Between 2006 and 2013, there were twice as many new instances of cancer overall, and by 2030, the number of new cases and fatal cases of breast cancer is predicted to increase by 80% and 50%, respectively (13). Despite the BSE is one of the secondary prevention of breast cancer and an important measure for early diagnosis of breast cancer, little is known about regular Breast self-examination. More documentation focuses on the general population, with little documentation of BSE practice among University students. The study aims to assess young adult women's knowledge, attitude, and practice regarding breast self-examination. The study has three specific objectives (I) To assess knowledge of self-breast examination among female undergraduates at Dodoma University (II) To examine female undergraduates’ attitudes towards self-breast examination at the University of Dodoma and (III) To determine BSE practice among female undergraduate's students at the University of Dodoma.

## Methodology

### Study area

The study will be carried out in the Dodoma region of Tanzania because it is a rapidly developing region with people from various cultural backgrounds. Tanzania has a total population of 61,741,120, with females (31,687,990) and males (30,053,130). Meanwhile, Dodoma Region is the capital city of Tanzania, found in the central zone of Tanzania, with 41,310 km^2^ and a total population of 3,085,625 people, female (1,572,865) and male (1,512,760) (14).

### Study design

An institutionally based analytical cross-sectional study will be conducted to examine female undergraduate students' knowledge, attitude, and practice regarding BSE.

### Study population

The population of this study is female undergraduate students studying at the two Universities located in Dodoma. Most of these students are young (18–22) and are directly from high schools.

### Inclusion criteria and exclusion criteria

Female undergraduate students studying at the Universities and aged 18 years and above years will be recruited into the study. Those who are unwilling to participate will be excluded from the study.

### Sample size calculation

Sample size calculation will be obtained using Kish Leslie's formula (15) as presented below.

$$n=\frac{Z^{2}\times p(1-p)}{d^{2}}$$

Whereby, *n *= Desired sample size, *Z* = confidence level at 95% (standard value of 1.96), *P* = estimated proportion of the respondents with a positive response towards BSE arbitrary proportion of 50%, and *d* = margin of error at 5% (standard value of 0.05).

$$\mathrm{Hence},n\;\;=\;\;\frac{{(1.96)}^{2}\;*\;0.5(1-0.5)}{\begin{array}{c} (0.05{)}^{2}\;\\ \;n\;=\;384\; \end{array}}$$

Therefore, the total sample size of the study will be 384 participants.

### Sampling technique

This study will utilize a stratified random sampling to conduct the study in two Universities. Simple random sampling at the Universities will be applied in selecting participants from each stratum (campus) thus respondents in each stratum will have an equal chance of being selected. The proportionate sampling will be applied to determine number of required samples at each stratum.

### Data collection procedure and data collection tools

The data will be collected from 30th May to June 2023 by a principal investigator and one research assistant, through a validated questionnaire. The research assistant will be trained on how to use the tool and how to collect data. The questionnaire about sociodemographics and knowledge of BSE is adopted from the previous study by Sayi (16), while the questionnaire to assess attitude and BSE practice is adopted from (17). The complete questionnaire has four sections; Section A: Sociodemographic characteristics, Section B: Knowledge level of breast self-examination, Section C: Attitude towards breast self-examination, and Section D: Breast self-examination practice. Refer to Appendix 1.

### Validity and reliability

The questionnaires adopted from the previous studies will be shared with experts to rank the effectiveness of tools and seek their comments for improvements. The pilot study will be conducted to pretest the tool, using 30% of actual sample size. If Cronbach's Alpa is 0.7 and above, the tools will be considered reliable. If Cronbach's Alpa is below 0.7, the Principle Component Analysis (PCA) will be utilized for factor analysis to modify weak variables.

### Data analysis plan

The data will be entered and analyzed in the Statistical Package for Social Sciences (SPSS) Version 25. Descriptive analysis (frequencies and percentages) will be used to analyze the participants’ sociodemographic characteristics, BSE knowledge, attitudes towards BSE, and BSE practices. Meanwhile, the inferential statistics will be used to compute the associations of variables through Cross-tabulation, Chi-square (*χ*^2^), and Regression analysis. The significant difference will be set at *P *= 0.05.

### Ethics approval and consent to participate

Ethical approval will be obtained from the University of Dodoma Institutional Research Review Committee (UDOM-IRREC). The introduction letter will be provided by the School of Nursing and Public Health of UDOM. The permission letter to conduct the study at the University of Dodoma will be provided by the council director of the Dodoma Municipality and the ward executive officer (WEO). The participants' confidentiality will be maintained by avoiding using participants' actual names and well-keeping filled questionnaires out of non-research team members. Each participant will complete the written informed consent before participating in the study. Participants will be free to participate and withdraw from the study at any time they feel to do so. The participants will be informed that there will be no compensation for their time.

## Discussion

### Knowledge of breast self-examination

The vast majority of previous studies demonstrate that there is a lack of knowledge on how to perform a thorough BSE. The research conducted in Bangladesh conducted among university students shows the overall mean score of total knowledge was 15 (SD = 3) out of 43, with an overall correct rate of 34% (18). Even though the majority of young women are aware of breast cancer, their knowledge of performing a self-breast check is still quite limited. A quasi-experimental carried out in Bangladesh reported low baseline awareness and knowledge of BSE (19). The campaigns about BSE should aim to promote knowledge and not awareness. The ongoing campaigns does not help women to understand deeper about BSE that's why women end up with awareness and inadequate knowledge. Despite the available studies on knowledge of BSE among University students, there is limited documentation concerning BSE knowledge among University students undertaking health related programs.

### Attitude on breast self-examination

An institutional-based cross-sectional study conducted among young adults attending family planning services revealed that 224 (53.2%) women believed that it would be simple to perform BSE, 293 (69.6%) women mentioned that BSE was not a sexual activity initiation, 179 (42.5%) women reported that it was inappropriate to touch their breasts, 73.9% women said that BSE is important for preventing breast cancer and 59.4% women want BSE because it does not result in a positive cancer test (20). The systematic scoping review that included 21 studies revealed that the majority of senior high school students in Nigeria (82.6%) and female nurses in Ghana and Ethiopia (59.2%) had positive attitudes toward BSE (21). Since the attitude is promising, more efforts need to focus on coverage to ensure everyone is educated on BSE. The coverage can be through media health education.

Despite the available studies on attitude of BSE among University students, there is limited documentation concerning attitude towards BSE among University students undertaking health related programs.

### BSE practice

A study conducted in Northwest Ethiopia in 2021 revealed that 17.4% of students studied had practiced BSE, which is associated with a lack of knowledge (22). Furthermore, the study among women attending family planning in southern Ethiopia revealed that 89 (21.1%) women have ever engaged in BSE, while 192 (57.8%) women have never practiced BSE because they believed they were healthy and others were afraid to be diagnosed with breast cancer (20). Meanwhile, the study in Turkey reported that 50% of students said they had performed BSE, and 33.3% reported having done so regularly. Only 13.3% of people reported performing a BSE routinely every month, compared to 55.5% who said they did it anytime they had the urge (23). Furthermore, 21% of Master's degree students reported practicing BSE (18). Mitigating the poor BSE practices, knowledge and attitude need to be considered factors. Despite the available studies on the practice of BSE among University students, there is limited documentation concerning BSE practice among University students undertaking health-related programs.

### Study implications

The finding will assist the policymakers and healthcare stakeholders in identifying issues that need to be addressed to lower breast cancer prevalence and mortality and it will help in the allocation of resources.

### Study limitation

Even though mammography is a recommended approach for detection of early stage of cancer, the practice of BSE will be assessed as it is within the capacity of an individual.

## Conclusion

The practice of BSE among women remains unsatisfactory, which is highly linked to the lack of BSE knowledge. The deficit knowledge is on how to perform BSE, the benefit of BSE, and the interval of doing BSE. Most of the women seem to possess a positive attitude towards BSE.

## Appendix

Section A: Sociodemographic characteristics

**Table:** Appendix 1 Questionnaire english version

Items	Responses
1. Age	(a) 18–20 (b) 21–25 (c) 26–30 (d) 31–50
2. Marital status	(a) Single (b) Married (c) Widow (d) Divorced/separated (e) Cohabiting
3. Year of your study	(a) First year (b) Second year (c) Third year (d) Fourth year (e) Fifth year
4. Religion	(a) Christian (b) Muslim (c) Others
5. Residence	(a) University campus (b) Outside the University campus (c) At home
6. What program are you studying?	

Section B: knowledge level of breast self-examination

1. What kind of things do you look for during self-examination yourself?

**Table:**

Items	Agree	Disagree
1. lumps in the breast		
2. Inverted nipples		
3. Nipples abnormal discharges		
4. Change in the breast skin textures such as redness, pain, and tenderness		
5. Change in the size and shape of the breasts		
6. Don’t know		

2. At what age should breast self-examination be practiced?

**Table:**

Items	Agree	Disagree
From 15 years		
From 20 years		
Above 30 years		
Others (specify)		
Don’t know		

3. What are the advantages of breast self-examination?

**Table:**

Items	Agree	Disagree
It helps to know the shape and size of the breast		
It is done to make it firm		
It helps to detect breast lumps earlier		
No advantage of BSE		
Don’t know		

4. If you notice a lump in your breast, what is the most likely thing you would do?

**Table:**

Items	Agree	Disagree
I go for treatment from a traditional healer		
Ignore		
Visit the clinic for further diagnosis		
Don’t know		

5. How often do you do the procedure of BSE?

**Table:**

Response	Agree	Disagree
Daily		
Weekly		
Monthly		
Yearly		
Never		
Don’t know		

Section C: attitude towards breast self-examination.

1. Is breast self-examination time-consuming?
  (a) Yes […]
  (b) No […]
2. Is breast self examination expensive?
  (a) Yes […]
  (b) No […]
3. Is it difficult to perfume breast self-examination?
  (a) Yes […]
  (b) No […]
4. Is breast self-examination embarrassing and unpleasant?
  (a) Yes […]
  (b) No […]

Section D: breast self-examination practice

1. Have you ever performed breast self-examination?
  (a) Yes […]
  (b) No […]
2. How often do you perform breast self-examination?
  (a) Everyday […]
  (b) Weakly […]
  (c) Monthly […]
  (d) Yearly […]
  (e) At random […]
3. What is the appropriate time to perform breast self-examination?
  (a) Before menstruation […]
  (b) During menstruation […]
  (c) Some days after menstruation […]
  (d) No particular time […]
4. How do you perform breast self-examination?
  (a) In front of the mirror […]
  (b) While showering […]
  (c) Lay down on the Bed […]
